# Account of the Latest Experiments on the Application of Galvanism, for Medical Purposes, Made in Germany

**Published:** 1803-10-01

**Authors:** 


### ACCOUNT *of the latest* EXPERIMENTS *on the* APPLICATION *of* GALVANISM, *for* MEDICAL PURPOSES, *made in* GERMANY.

[ Continued from p. 62. ]

IN applying Galvanism for the cure of amaurosis, Dr. Martens made use of the following methods: He conducted it by means of two directors of brass wire, which were insulated to the nervous branches that issue above and below the eye; but finding this method not to answer its purpose, he took two copper plates, which were on one side a little excavated, and flat on the other; he combined them with a string which he fastened in the rings made on the flat side of the plates, and thus tied them to the eyelids, after he had laid under the plates round pieces of cloth, which had been previously dipped in pure water; the conducting chains were afterwards applied to the rings on the back of the plates. This manner of applying Galvanism however was soon left by the author, when he perceived that after each experiment the conjunctiva became red and inflamed; in lieu of which he adopted the following method: He ordered the patient to take in his mouth a silver spatula, to which the conducting chain had been adapted, adapted, and having moistened the eyelids, he applied to them a double armed conductor, which was attached to the other chain. In complete amaurosis, he thinks it advisable to put a blister on the processus mastoideus; and the place having been deprived of the cuticula, he applied one conductor to it, while the other was held to the eye of the same side. Dr. M. mentions a case of rheumatic tooth-ach, which was instantly removed by applying on each side of the gums the two Galvanic conductors. The most proper time for the application of Galvanism is the forenoon, the body being then more sensible and fit to receive the impression of the Galvanic stimulus. He likewise cautions the patients who use the Galvanism in difficulty of hearing and deafness, against drinking wine, at least he advises to abstain from it during the day when they are galvanised. Dr. M. farther endeavours to determine the indications for applying the Galvanic agens, which according to him are the following: 1. Diminished activity of the cutaneous organ, suppressed transpiration, or any other obstruction in the cutaneous vessels and the tela cellulosa, &c. hence its great use in rheumatic complaints. 2. Diminished activity in the circulation of the blood, extravasations and obstructions in the blood vessels, &c. whence we may derive great advantage from the application of Galvanism in wens and bronchocele, &c. 3. Torpid state of the nervous system.

The indications which are to be followed in single morbid states, in which Galvanism may be employed, are thus stated by the author. 1. In deafness and difficulty of hearing, particularly in young healthy persons, and if the complaint is not too much rooted; farther, in deafness originating from a rheumatic cause, from a paralytic state of the auditory nerve remaining after apoplectic fits, &c. Contra-indications in these cases, are congestions of blood towards the head in plethoric subjects, in which a strong tinkling of the ears generally takes place, running of the eyes, hereditary deafness, &c. paralyses which are not too inveterate. 3. Amaurosis, that species particularly which originates in a paralytic state of the optic nerve. 4. Rheumatic complaints, arthritis, ischias, tooth-ach, in all which complaints Galvanism has been frequently employed with success. 5. It is likewise a very good stimulus in asphyxia or apparent death.

On drawing a parallel between Galvanism and Electricity, Dr. Martens finds them to differ in the following points: 1. Galvanism is stronger, more penetrating and efficacious, its stimulus being at the same time more permanent. 2. Its action is generally limited to the part part on which it is directed. 3. Galvanism seems not to have the power of attracting light bodies, as we observe in electricity. 4. It seems not to be increased by insulating, as is the case with electricity. For medical purposes the application of Galvanism is undoubtedly preferable to that of electricity, as it is more convenient, and its action more certain and efficacious than electricity. There are finally added, some cases in which Galvanism was successfully employed by a practitioner, of which we think the first only worthy to be mentioned, where a paralysis of one side of the face was cured by means of the Galvanic agens after the fruitless application of other remedies.

Dr. Marcus, of Bamberg, likewise communicates several experiments which were made with the medicinal application of Galvanism at the great hospital of Bamberg. It was used in several cases of paralysis, as hemiplegia of the left side, paralysis of the right and of the left arm, in the last of which cases a complete cure was effected merely through the medium of Galvanism; farther, in several cases of deafness and difficulty of hearing, where it generally produced considerable relief. He employed it also in the following cases. 1. A violent head-ach which had remained after a remitting fever, and which would not yield to any remedy, was removed by the application of the Galvanic stimulus. The pain began to cease during the first experiment, when the temples, the forehead and the neck were moderately galvanised. Two or three days after, as the head-ach had returned, the experiment was repeated, by which it was entirely cured. 2. A sciatica, which had resisted all remedies for more than two months was entirely cured by Galvanism, after its application had been repeated for eleven days, one after another. 3. Epilepsy, of which three cases are related, where the paroxysm immediately ceased on bringing the patient in combination with the Galvanic battery by applying one hand to the negative pole, while the other was held on the positive. Besides this, Dr. M. orders the patient to be galvanised at the time when he is free from his disease, and he found in one case the paroxysm was postponed. He recommends to apply the Galvanism to the back bone, where the nerves of the neck, of the back, and of the lumbar region issue.

In Professor Loder's Journal for Surgery, we find also some accounts of the successful application of Galvanism in several diseases, particularly in dimness of sight, &c.

Dr. Hellwag, of Eutin, in the bishoprick of Lubeck, has communicated his experiments on applying Galvanism ism for medicinal purposes, in a small work entitled, "Erfahrungen uber die Heitkraste des Galvanismus;" i. e. Observations and Experiments on the Medicinal Virtues of Galvanism. The first experiment was made on a boy five years of age, who was deaf and dumb, though he seemed still to hear very strong sounds. The battery of which he made use was constructed of crown pieces and zinc-plate with round pieces of paste-board soaked in salt water. One of the conductors being applied to one ear, the other ear was touched with the other conductor, moving it to the ear and from it alternately. A battery of 20 strata was thought sufficient; but when its action seemed to decrease, the number of strata was raised to forty. At the end of the two conductors, round pieces of sponge soaked in salt water were afterwards fastened, which were introduced into the ears. The operation lasted generally from ten to twenty minutes. The experiments were thus continued for about a fortnight, before any effect could be perceived; but about this time the patient began to hearken to moderate sounds with visible pleasure, and even turned round when he was gently called by his name; he seemed to pay attention to every new sound which he perceived, and by degrees he could even hear a watch go at the distance of ten inches; in this way his audition gradually improved, and he learned to pronounce letters and words, though but imperfectly; but not finding it attended with further benefit, the experiments were discontinued. The success however of this cure induced Dr. Hellwag to make farther trials with the medical application of Galvanism, for which purpose he constructed a new battery, taking plates of metal, of which printing types are cast, instead of the silver crown-pieces, and in place of the salt water he chose the gall as a wet conductor, which he found extremely efficacious. For better convenience the battery was placed horizontally. The conductors were brass wires, at the end of which he fastened small sponges, which he could conveniently introduce into the ear. Dr. H. then relates six cases of deafness, in which he employed Galvanism. A youth, of eighteen years, who had lost the power of hearing by a scrophulous ulcer in one ear, and the audition in the other had suffered considerably, was galvanized with seeming success, but shortly after he relapsed into his former state. Another patient, twenty years of age, who was born deaf, and whose organs of hearing were touched only by certain sounds, as the barking of a dog, the sound of a flute or harp, had been galvanized about a fortnight for half an hour every day, when his ear became sensible to other sounds, as even to hear the noise which a watch makes when going. A similar success was observed in a girl, sixteen years of age, who was nearly deaf before. A young man was frequently affected with difficulty of hearing, which had often yielded to different external remedies. As his complaint returned, Dr. H. began to try on him the Galvanism, and having been under his care for some days, the patient suddenly raised himself during the experiment, as he felt a violent head-ach, attended with strong tinkling in the ears, which having gradually decreased, he perfectly recovered his hearing. In another man, sixty-eight years of age, who after a retrogade exanthema, had been afflicted with dimness of sight and difficulty of hearing, was considerably relieved by the use of Galvanism. A girl, nine years of age, was nearly cured of a difficulty of hearing, which occurred after a violent otalgia. Dr. Hellwag likewise employed the Galvanism in other diseases, of which he relates five cases, partly paralytic diseases, and two cases of amenorrhœa; but no remarkable effect could be observed from the application of that stimulus.

At the end of Dr. Hellwag's publication are annexed some observations and experiments with the medical applications of Galvanism, made by Dr. Jacobi, which he owns were not attended with such a degree of success as other medical writers have boasted of. The author then enlarges on the causes of the unsuccessful application of Galvanism, which partly is owing to the improper application, partly to a want of tone in the nervous and muscular fibres, which he thinks must in general be present to secure the success of the Galvanic operation; he therefore advises to employ a tonic method of cure at the same time with Galvanism, as cold baths, sea baths, steel, bark, &c.

